# Laparoscopic repair of ureter damaged during laparoscopic hysterectomy: Presentation of two cases

**DOI:** 10.4274/tjod.34270

**Published:** 2017-09-30

**Authors:** Murat Api, Ayşen Boza, Semra Kayataş, Barış Boza

**Affiliations:** 1 İstanbul Medipol University Faculty of Medicine, Department of Obstetrics and Gynecology, İstanbul, Turkey; 2 Vehbi Koç Foundation American Hospital, Women’s Health Center Assisted Reproduction Unit, İstanbul, Turkey; 3 University of Health Sciences, Zeynep Kamil Women and Children’s Health Training and Research Hospital, İstanbul, Turkey

**Keywords:** Ureter, Injury, complication, laparoscopy

## Abstract

Ureter injuries are uncommon but dreaded complications in gynecologic surgery and a frequent cause of conversion to laparotomy. Recently, a few papers reported the repair of gynecologic ureteral injuries using laparoscopy with encouraging results. In these case reports, we aimed to present two laparoscopically repaired ureter injuries during total laparoscopic hysterectomies (TLH). In the first case, the ureter was transected during the dissection of the cardinal ligament, approximately 7 to 8 cm distal to the ureterovesical junction (UVJ), and in the second case, it was damaged approximately 10 cm distal to the UVJ. Both transections were identified during surgery. The injured ureter was repaired without converting to laparotomy or additional trocar insertion. Ureteroureterostomy was performed in both cases uneventfully. Although ureteric injury is a rare complication during TLH, it can be managed by the same surgeon laparoscopically during the same procedure.

## INTRODUCTION

Urologic complications during gynecologic surgery are uncommon but dreadful. The reported incidence of ureteral injury related to gynecologic surgery varies between 2.5% and 12.1%^(1)^. Pelvic ureters are retroperitoneal structures that run from the renal pelvis to the bladder that can be injured during pelvic surgery at any point along their distal course. However, the approach of hysterectomy plays a role in the variation of incidence in total laparoscopic hysterectomies (LHs) (TLHs) compared with the abdominal and vaginal approach; the reported odds ratio for urinary tract injuries was 2.41 and 3.69, respectively^(2)^. Although the risk of ureter injury significantly decreases with the increasing experience of surgeons, ureter injuries also occur in the hands of experienced gynecologists because the indication for TLH is expanding and the difficulty of the operation is increasing. The question is whether complications of laparoscopic procedures such as ureteral injury could be managed successfully and effectively without converting to laparotomy. We report successful immediate primary laparoscopic repair of two cases of ureter transection during TLH and discuss possible causes that lead to ureter injury and make recommendations for prevention.

## CASE REPORT

**Figure 1:**
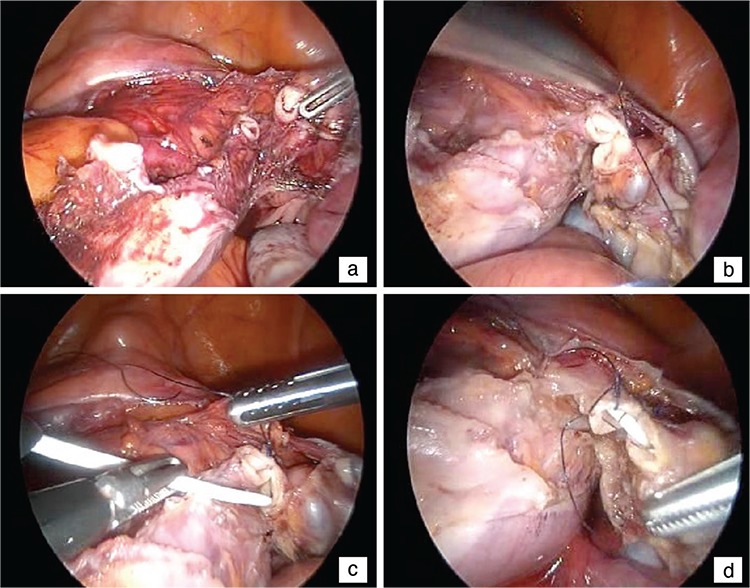
The surgical technique of laparoscopic ureter injury is shown. The transected edges of the ureter at the area of cardinal ligament are seen (a). First, the proximal and distal ureter edges are sutured at the 6 o’clock position (b), then the ureteral stent is inserted in the ureter (c) and the anastomosis is completed using sutures placed at the 9, 12 and 3 o’clock positions (d)

A patient aged 48 years underwent TLH and bilateral salpingo-oophorectomy (BSO) due to abnormal uterine bleeding that was unresponsive to medical therapy. She had no previous history of abdominal surgery or any kind of disease causing pelvic adhesions. On exploration, the uterus was of 8 weeks’ pregnancy in size, the bilateral adnexa were normal, and no pelvic adhesions or distortion of anatomy was observed. A four-trocar technique (out of main trocar, two at the left side and one at the right side) and Hohl manipulator (manufactured by Karl Storz, Germany) was used. The TLH was uneventful until the area of the cardinal ligaments (CLs). The ureter was transected with the Ligasure™ (Valleylab, Boulder, CO, USA) during the dissection of the right CL, 7 to 8 cm distant from the ureterovesical junction (UVJ) (Figure 1a). The transection was identified during the dissection and immediate repair was performed peroperatively without converting to laparotomy. The proximal and distal ureteral segments were identified, then sutured at the 6 o’clock position for approximation (Figure 1b). A double J ureteral stent was taken to the abdomen through the 5-mm lateral trocar and inserted in the ureter (Figure 1c). After the stent application, 3/0 Vicryl sutures were placed at the 9, 12, and 3 o’clock positions (Figure 1d). A Foley catheter remained in the bladder and a drainage tube in the abdomen to prevent possible urinoma formation and to control bleeding.

**Figure 2:**
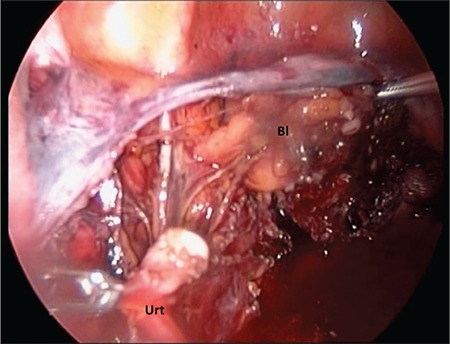
The transected edges of the ureter at the level of left cardinal ligament were seen Urt: Ureter, Bl: Bladder

In the second case, a woman aged 59 years underwent TLH and BSO due to simple endometrial hyperplasia with atypia. TLH, BSO, and frozen pathology were planned with the suspicion of malignancy. Laparoscopy was performed using the same technique as in the first case. Frozen section was reported in favor of benign pathology. During the dissection of the left CL, the ureter was damaged approximately 10 cm distant from UVJ with the Halo™ bipolar cutting forceps (Gyrus ACMI, Olympus) (Figure 2). The injured ureter was noticed during the dissection and repaired without converting to laparotomy or additional trocar insertion, similar to the first case. The duration of the ureteral repairs from the time of the injury to the end of the last anastomosis suture were 55 and 40 minutes for the first and second cases, respectively. Estimated blood loss during the ureteral repairs was negligible. The vesical catheters were withdrawn on 7 days day postoperatively and the ureteral stents were removed cystoscopically on the 21^st^ day. We did not use prophylactic antibiotics due to prolonged catheterization. Patients were closely monitored after discharge weekly for the first three weeks, then monthly up to 10-12 months using serial urinary system ultrasonography. Neither patient had any pelvicalyceal ectasia or visible ureters. The patients’ recoveries were uneventful with normal kidney and urinary function. Written informed consent was obtained by both patients.

## DISCUSSION

Ureter injuries can result from pelvic dissection or due to thermal injury by excessive use of energy adjacent to the ureter. In a retrospective review of 165 patients with iatrogenic ureteral injuries over a 20-year span, endourologic procedures were responsible for most iatrogenic injuries with 42%, and gynecologic and general surgery procedures were 34% and 24%, respectively^(3)^. Urinary tract injuries during LH are reported to be more frequent than in abdominal or vaginal hysterectomies. The higher risk in LH compared with the abdominal approach is due to the use of electrocoagulation of uterine vessels during laparoscopic procedures^(4)^. Extensive electrocoagulation of uterine vessels and CLs near the ureter increases the risk of ureter injuries.

Intraoperative detection of ureter injuries has been reported in only 5-13% of cases^(3,4)^. Among the acute injuries of the urinary tract, ureter injuries are most difficult to recognize as there often may be few or with no symptoms^(1)^. Intraoperative iatrogenic ureteral injuries, if recognized during the procedure, should be repaired at that setting^(5)^. In our cases, the transected edges of the ureter were seen without any urine leakage during the dissection of the CLs.

Approximately 90% of the trauma to the ureter occurs in the lower portion, which extends from the inferior border of the sacroiliac joint to the UVJ^(6)^. Transection injuries are repaired depending on the severity of the injury and approximation to the UVJ. If the ureteral injury is more than 6 cm distant from the UVJ, a primary ureteral anastomosis is performed. Primary ureteroureterostomy is the optimal technique when the anastomosis can be performed without tension and if the initial approach was laparoscopic^(7)^. Laparoscopic ureteroureterostomy was preferred in our cases because the mid-ureter was transected approximately 8 cm and 10 cm distant from the UVJ, respectively.

Laparoscopic ureteral repair is technically feasible and an alternative to open repair for immediate, early, and delayed diagnosis of ureteral injury. In the literature, although long-term outcome of laparoscopic repair is limited to small series and case reports, the results are excellent if the diagnosis and repair is performed at the time of injury^(8)^. A delay in diagnosis worsens prognosis because of infection, hydronephrosis, abscess, and fistula formation. No postoperative complication occurred in our cases, the patients have continued their lives with normal kidney function and no urinary incontinence for 10 and 12 months follow-up, respectively.

Recommended techniques for reducing the risk of urinary tract injuries were agreed upon by experts in a Delphi consensus procedure: routine use of uterine manipulator, coagulation of uterine vessels close to the uterus with a perpendicular approach from the ipsilateral side, and visualization or dissection of the ureter in the case of distorted anatomy^(9)^. Intraoperative routine use of cystoscopy was proposed to assess the ureter flow as a part of all TLHs. Cystoscopy during TLH is well tolerated and can reassure surgeons of immediate urinary tract injuries; however, it is an additional time-consuming procedure that is not required in most patients. Cystoscopy during TLHs was postulated to be cost effective if the rate of ureter injury exceeded 2%^(10)^.

In the case of ureteral injury during laparoscopic gynecologic surgery, either a gynecologist or urologist who is experienced in suturing and knot-tying techniques can successfully perform a primary laparoscopic repair of the ureter. Visualization of the course of ureters at the beginning or end of the operation may prevent complications of the urinary tract and possible delayed diagnosis of ureteral injuries.
